# {μ-*trans*-*N*,*N*′-Bis[(diphenyl­phosphan­yl)meth­yl]benzene-1,4-diamine-κ^2^ *P*:*P*′}bis­{(acetonitrile-κ*N*)[dipyrido[3,2-*a*:2′,3′-*c*]phenazine-κ^2^ *N* ^4^,*N* ^5^]copper(I)} bis­(tetra­fluoridoborate)

**DOI:** 10.1107/S1600536809031754

**Published:** 2009-08-19

**Authors:** Ting-Hong Huang, Xuan-Feng Jiang, Liu-Cheng Gui, Xiu-Jian Wang, Zhong-Min Cen

**Affiliations:** a School of Chemistry and Chemical Engineering, Guangxi Normal University, Guilin 541004, People’s Republic of China

## Abstract

In the centrosymmetric dinuclear title compound, [Cu_2_(C_2_H_3_N)_2_(C_18_H_10_N_4_)_2_(C_32_H_30_N_2_P_2_)](BF_4_)_2_, the Cu^I^ centre is coordinated by two N atoms from a dipyridophenazine ligand, one P atom from an *N*,*N*′-bis­[(diphenyl­phosphan­yl)meth­yl]benzene-1,4-diamine (bpbda) ligand, and one N atom from an acetonitrile mol­ecule in a distorted tetra­hedral geometry. The bpbda ligand, lying on an inversion center, bridges two Cu^I^ centres into a Z-shaped complex. Intra­molecular π–π inter­actions between the dipyridophenazine ligand and the benzene ring of the bpbda ligand are observed [centroid–centroid distance = 3.459 (3) Å]. The crystal structure also involves inter­molecular π–π inter­actions between the dipyridophenazine ligands [centroid–centroid distance = 3.506 (3) Å], which lead to a one-dimensional supra­molecular structure.

## Related literature

For general background to π–π inter­actions in chemistry and biochemistry, see: Aucott *et al.* (2002 ▶); Chipot *et al.* (1996 ▶); Saenger (1984 ▶); Wang *et al.* (2008 ▶); Waters (2002 ▶).
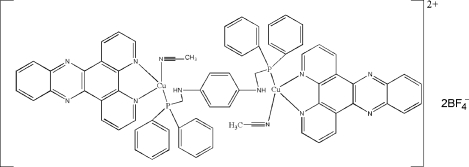


## Experimental

### 

#### Crystal data

[Cu_2_(C_2_H_3_N)_2_(C_18_H_10_N_4_)_2_(C_32_H_30_N_2_P_2_)](BF_4_)_2_
*M* *_r_* = 1451.93
Triclinic, 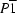

*a* = 12.1074 (3) Å
*b* = 12.3354 (2) Å
*c* = 12.6262 (3) Å
α = 84.905 (1)°
β = 68.250 (1)°
γ = 66.732 (1)°
*V* = 1605.35 (6) Å^3^
*Z* = 1
Mo *K*α radiation
μ = 0.79 mm^−1^
*T* = 293 K
0.32 × 0.24 × 0.20 mm

#### Data collection

Rigaku Mercury CCD diffractometer
Absorption correction: multi-scan (*CrystalClear*; Rigaku, 2005 ▶) *T*_min_ = 0.786, *T*_max_ = 0.858
15862 measured reflections
7288 independent reflections
6153 reflections with *I* > 2σ(*I*)
*R*_int_ = 0.020

#### Refinement

*R*[*F* ^2^ > 2σ(*F* ^2^)] = 0.041
*wR*(*F* ^2^) = 0.119
*S* = 1.08
7288 reflections
443 parameters
H-atom parameters constrained
Δρ_max_ = 0.80 e Å^−3^
Δρ_min_ = −0.58 e Å^−3^

### 

Data collection: *CrystalClear* (Rigaku 2005 ▶); cell refinement: *CrystalClear*; data reduction: *CrystalClear*; program(s) used to solve structure: *SHELXS97* (Sheldrick, 2008 ▶); program(s) used to refine structure: *SHELXL97* (Sheldrick, 2008 ▶); molecular graphics: *SHELXTL* (Sheldrick, 2008 ▶); software used to prepare material for publication: *SHELXTL*.

## Supplementary Material

Crystal structure: contains datablocks I, global. DOI: 10.1107/S1600536809031754/hy2208sup1.cif

Structure factors: contains datablocks I. DOI: 10.1107/S1600536809031754/hy2208Isup2.hkl

Additional supplementary materials:  crystallographic information; 3D view; checkCIF report

## Figures and Tables

**Table 1:** Selected bond lengths (Å)

Cu1—N2	2.0828 (19)
Cu1—N3	2.0628 (18)
Cu1—N6	2.013 (2)
Cu1—P2	2.1883 (6)

**Fig. 1.**
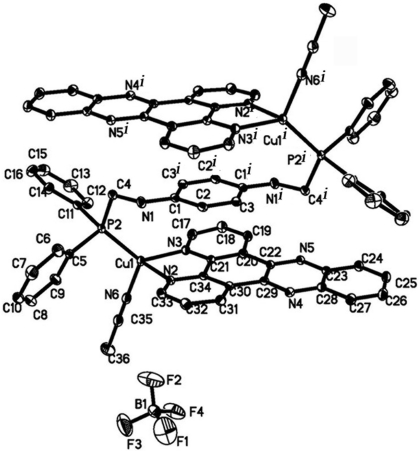
The molecular structure of the title complex. Displacement ellipsoids are drawn at the 30% probability level. H atoms have been omitted for clarity. [Symmetry code: (i) -x, 1 - y, -z.]

## supplementary crystallographic information

### Comment

π–π Stacking interactions between aromatic systems have been reported in many fields of chemistry and biochemistry (Aucott *et al.*, 2002; Chipot *et al.*, 1996). They play an important role in the structures of biological macromolecules (Saenger, 1984). For example, they are exploited for the intercalation of drugs into DNA (Waters, 2002). Information on the structures of copper(I) compouds with π–π stacking Interactions, which lead to infinite linear chain, continues to be reported (Wang *et al.*, 2008). In the title binuclear copper(I) complex, the *N*,*N*'-bis[(diphenylphosphanyl)methyl]benzene-1,4-diamine ligand, lying on an inversion center, links two Cu^I^ atoms through the P atoms (Fig.1). The Cu^I^ atom has a distorted tetrahedral coordination geometry. The structure involves intra- and intermolecular π–π interactions with centroid–centroid distances of 3.459 (3) and 3.506 (3) Å, respectively. The intermolecular π–π interactions connect the complex molecules into a one-dimensional supramolecular structure.

### Experimental

CuBF_4_.4CH_3_CN (0.066 g, 0.2 mmol) was added with stirring to a solution of dipyrido[3,2 - a:2',3'-c]phenazine (0.056 g, 0.2 mmol) and *N*,*N*'-bis[(diphenylphosphanyl)methyl]benzene-1,4-diamine (0.050 g, 0.10 mmol) in DMF(10 ml). The resulting solution was allowed to stir for 2 h. Then by slow diffusion of diethyl ether into the solution, block red crystals were deposited in 6 d (yield: 60%).

### Refinement

H atoms were positioned geometrically and refined as riding atoms, with C—H = 0.93 (aromatic), 0.97 (CH_2_), and 0.96 (CH_3_) Å and N—H = 0.86 Å, and with *U*_iso_(H) = 1.2(1.5 for methyl)*U*_eq_(C,*N*).

### Crystal data

**Table:**

[Cu_2_(C_2_H_3_N)_2_(C_18_H_10_N_4_)_2_(C_32_H_30_N_2_P_2_)](BF_4_)_2_	*Z* = 1
*M**_r_* = 1451.93	*F*(000) = 742
Triclinic, *P*1	*D*_x_ = 1.502 Mg m^−^^3^
Hall symbol: -P 1	Mo *K*α radiation, λ = 0.71073 Å
*a* = 12.1074 (3) Å	Cell parameters from 12749 reflections
*b* = 12.3354 (2) Å	θ = 2.6–27.5°
*c* = 12.6262 (3) Å	µ = 0.79 mm^−^^1^
α = 84.905 (1)°	*T* = 293 K
β = 68.250 (1)°	Block, red
γ = 66.732 (1)°	0.32 × 0.24 × 0.20 mm
*V* = 1605.35 (6) Å^3^	

### Data collection

**Table:**

Rigaku Mercury CCD diffractometer	7288 independent reflections
Radiation source: fine-focus sealed tube	6153 reflections with *I* > 2σ(*I*)
graphite	*R*_int_ = 0.020
ω scans	θ_max_ = 27.5°, θ_min_ = 3.1°
Absorption correction: multi-scan (*CrystalClear*; Rigaku, 2005)	*h* = −15→15
*T*_min_ = 0.786, *T*_max_ = 0.858	*k* = −14→16
15862 measured reflections	*l* = −16→16

### Refinement

**Table:**

Refinement on *F*^2^	Primary atom site location: structure-invariant direct methods
Least-squares matrix: full	Secondary atom site location: difference Fourier map
*R*[*F*^2^ > 2σ(*F*^2^)] = 0.041	Hydrogen site location: inferred from neighbouring sites
*wR*(*F*^2^) = 0.119	H-atom parameters constrained
*S* = 1.08	*w* = 1/[σ^2^(*F*_o_^2^) + (0.0641*P*)^2^ + 1.098*P*] where *P* = (*F*_o_^2^ + 2*F*_c_^2^)/3
7288 reflections	(Δ/σ)_max_ = 0.001
443 parameters	Δρ_max_ = 0.80 e Å^−^^3^
0 restraints	Δρ_min_ = −0.58 e Å^−^^3^

### Fractional atomic coordinates and isotropic or equivalent isotropic displacement parameters (Å^2^)

**Table:**

	*x*	*y*	*z*	*U*_iso_*/*U*_eq_	
Cu1	0.10933 (3)	0.22834 (2)	0.22835 (2)	0.02592 (10)	
P2	−0.08284 (5)	0.22819 (5)	0.26979 (4)	0.02005 (12)	
N1	−0.17869 (19)	0.45511 (16)	0.19707 (16)	0.0271 (4)	
H1A	−0.2264	0.5113	0.2499	0.033*	
N2	0.09991 (17)	0.39591 (17)	0.25741 (15)	0.0243 (4)	
N3	0.24317 (17)	0.24169 (16)	0.07427 (16)	0.0235 (4)	
N4	0.28443 (17)	0.67402 (16)	0.06715 (16)	0.0235 (4)	
N5	0.43829 (17)	0.50979 (16)	−0.12465 (15)	0.0229 (4)	
N6	0.2192 (2)	0.1156 (2)	0.3096 (2)	0.0385 (5)	
C1	−0.0878 (2)	0.47431 (18)	0.09971 (18)	0.0217 (4)	
C2	−0.0740 (2)	0.58216 (18)	0.09225 (18)	0.0236 (4)	
H2A	−0.1231	0.6381	0.1539	0.028*	
C3	0.0115 (2)	0.60781 (18)	−0.00522 (19)	0.0239 (4)	
H3A	0.0181	0.6807	−0.0077	0.029*	
C4	−0.1965 (2)	0.34561 (19)	0.21331 (19)	0.0252 (4)	
H4A	−0.1872	0.3157	0.1407	0.030*	
H4B	−0.2841	0.3614	0.2657	0.030*	
C5	−0.1659 (2)	0.24715 (19)	0.42484 (18)	0.0233 (4)	
C6	−0.2826 (3)	0.3388 (2)	0.4826 (2)	0.0369 (5)	
H6A	−0.3246	0.3960	0.4420	0.044*	
C7	−0.3369 (3)	0.3449 (3)	0.6016 (2)	0.0450 (7)	
H7A	−0.4155	0.4060	0.6399	0.054*	
C8	−0.2757 (3)	0.2619 (3)	0.6627 (2)	0.0456 (7)	
H8A	−0.3124	0.2669	0.7421	0.055*	
C9	−0.1597 (3)	0.1711 (3)	0.6061 (2)	0.0435 (6)	
H9A	−0.1184	0.1141	0.6473	0.052*	
C10	−0.1044 (3)	0.1641 (2)	0.4884 (2)	0.0346 (5)	
H10A	−0.0252	0.1032	0.4511	0.042*	
C11	−0.1028 (2)	0.09916 (18)	0.23379 (18)	0.0225 (4)	
C12	0.0021 (2)	0.0079 (2)	0.1606 (2)	0.0340 (5)	
H12A	0.0829	0.0121	0.1315	0.041*	
C13	−0.0124 (3)	−0.0894 (2)	0.1304 (3)	0.0458 (7)	
H13A	0.0584	−0.1500	0.0808	0.055*	
C14	−0.1316 (3)	−0.0966 (2)	0.1738 (3)	0.0422 (6)	
H14A	−0.1408	−0.1629	0.1547	0.051*	
C15	−0.2375 (3)	−0.0056 (2)	0.2456 (2)	0.0394 (6)	
H15A	−0.3182	−0.0101	0.2737	0.047*	
C16	−0.2240 (2)	0.0923 (2)	0.2760 (2)	0.0314 (5)	
H16A	−0.2955	0.1535	0.3244	0.038*	
C17	0.3083 (2)	0.16616 (19)	−0.0179 (2)	0.0285 (5)	
H17A	0.2994	0.0942	−0.0134	0.034*	
C18	0.3884 (2)	0.1896 (2)	−0.1201 (2)	0.0323 (5)	
H18A	0.4305	0.1351	−0.1829	0.039*	
C19	0.4051 (2)	0.2946 (2)	−0.12743 (19)	0.0266 (4)	
H19A	0.4584	0.3120	−0.1954	0.032*	
C20	0.3411 (2)	0.37443 (18)	−0.03170 (18)	0.0221 (4)	
C21	0.25879 (19)	0.34513 (18)	0.06730 (17)	0.0203 (4)	
C22	0.35395 (19)	0.48708 (18)	−0.03267 (17)	0.0203 (4)	
C23	0.4471 (2)	0.61545 (19)	−0.12338 (18)	0.0228 (4)	
C24	0.5343 (2)	0.6453 (2)	−0.2205 (2)	0.0284 (5)	
H24A	0.5846	0.5925	−0.2846	0.034*	
C25	0.5442 (2)	0.7513 (2)	−0.2196 (2)	0.0319 (5)	
H25A	0.6007	0.7708	−0.2836	0.038*	
C26	0.4696 (2)	0.8321 (2)	−0.1225 (2)	0.0321 (5)	
H26A	0.4793	0.9033	−0.1228	0.039*	
C27	0.3836 (2)	0.8074 (2)	−0.0283 (2)	0.0293 (5)	
H27A	0.3344	0.8619	0.0346	0.035*	
C28	0.3696 (2)	0.69847 (19)	−0.02664 (19)	0.0233 (4)	
C29	0.27640 (19)	0.57019 (18)	0.06501 (17)	0.0208 (4)	
C30	0.1861 (2)	0.54033 (19)	0.16478 (17)	0.0213 (4)	
C31	0.1007 (2)	0.6216 (2)	0.25919 (19)	0.0272 (4)	
H31A	0.1013	0.6966	0.2607	0.033*	
C32	0.0160 (2)	0.5884 (2)	0.3496 (2)	0.0321 (5)	
H32A	−0.0428	0.6413	0.4122	0.039*	
C33	0.0201 (2)	0.4748 (2)	0.34555 (19)	0.0304 (5)	
H33A	−0.0359	0.4526	0.4077	0.037*	
C34	0.18047 (19)	0.42966 (19)	0.16690 (17)	0.0214 (4)	
C35	0.2723 (3)	0.0633 (3)	0.3657 (3)	0.0412 (6)	
C36	0.3417 (4)	−0.0034 (4)	0.4389 (3)	0.0687 (11)	
H36A	0.4198	0.0095	0.4199	0.103*	
H36B	0.3627	−0.0862	0.4274	0.103*	
H36C	0.2882	0.0225	0.5174	0.103*	
B1	0.3624 (3)	0.2824 (3)	0.5497 (3)	0.0415 (7)	
F1	0.3397 (4)	0.3829 (3)	0.5988 (3)	0.1497 (16)	
F2	0.2814 (2)	0.2991 (4)	0.4946 (2)	0.1521 (18)	
F3	0.3415 (4)	0.2126 (3)	0.6392 (3)	0.1330 (13)	
F4	0.4840 (2)	0.2329 (3)	0.47575 (19)	0.0965 (9)	

### Atomic displacement parameters (Å^2^)

**Table:**

	*U*^11^	*U*^22^	*U*^33^	*U*^12^	*U*^13^	*U*^23^
Cu1	0.02530 (15)	0.03051 (16)	0.02609 (16)	−0.01641 (12)	−0.00980 (11)	0.01035 (11)
P2	0.0219 (3)	0.0217 (3)	0.0183 (3)	−0.0115 (2)	−0.0070 (2)	0.00549 (19)
N1	0.0339 (10)	0.0211 (8)	0.0254 (10)	−0.0120 (8)	−0.0094 (8)	0.0054 (7)
N2	0.0240 (9)	0.0328 (10)	0.0186 (9)	−0.0149 (8)	−0.0074 (7)	0.0058 (7)
N3	0.0220 (8)	0.0247 (9)	0.0244 (9)	−0.0103 (7)	−0.0085 (7)	0.0052 (7)
N4	0.0230 (9)	0.0243 (9)	0.0239 (9)	−0.0097 (7)	−0.0090 (7)	0.0021 (7)
N5	0.0222 (8)	0.0267 (9)	0.0211 (9)	−0.0115 (7)	−0.0080 (7)	0.0044 (7)
N6	0.0364 (11)	0.0447 (12)	0.0473 (13)	−0.0256 (10)	−0.0234 (10)	0.0239 (10)
C1	0.0260 (10)	0.0210 (9)	0.0223 (10)	−0.0100 (8)	−0.0137 (8)	0.0071 (8)
C2	0.0301 (11)	0.0208 (10)	0.0224 (10)	−0.0094 (9)	−0.0133 (9)	0.0030 (8)
C3	0.0318 (11)	0.0188 (9)	0.0266 (11)	−0.0118 (9)	−0.0154 (9)	0.0055 (8)
C4	0.0257 (10)	0.0267 (10)	0.0262 (11)	−0.0122 (9)	−0.0122 (9)	0.0092 (8)
C5	0.0277 (10)	0.0276 (10)	0.0190 (10)	−0.0173 (9)	−0.0067 (8)	0.0024 (8)
C6	0.0361 (13)	0.0379 (13)	0.0299 (13)	−0.0126 (11)	−0.0062 (10)	−0.0005 (10)
C7	0.0399 (14)	0.0537 (17)	0.0315 (14)	−0.0204 (13)	0.0034 (11)	−0.0143 (12)
C8	0.0491 (16)	0.079 (2)	0.0187 (12)	−0.0401 (16)	−0.0059 (11)	−0.0017 (12)
C9	0.0506 (16)	0.0622 (18)	0.0270 (13)	−0.0295 (15)	−0.0184 (12)	0.0127 (12)
C10	0.0368 (13)	0.0413 (13)	0.0247 (12)	−0.0149 (11)	−0.0114 (10)	0.0063 (10)
C11	0.0289 (10)	0.0214 (9)	0.0192 (10)	−0.0110 (9)	−0.0102 (8)	0.0037 (8)
C12	0.0303 (12)	0.0300 (12)	0.0368 (13)	−0.0082 (10)	−0.0103 (10)	−0.0012 (10)
C13	0.0467 (16)	0.0277 (12)	0.0545 (18)	−0.0031 (12)	−0.0192 (14)	−0.0102 (12)
C14	0.0585 (17)	0.0266 (12)	0.0513 (17)	−0.0180 (12)	−0.0291 (14)	0.0012 (11)
C15	0.0446 (15)	0.0397 (14)	0.0466 (16)	−0.0259 (12)	−0.0208 (12)	0.0062 (12)
C16	0.0318 (12)	0.0314 (12)	0.0311 (12)	−0.0154 (10)	−0.0076 (10)	−0.0011 (9)
C17	0.0316 (11)	0.0220 (10)	0.0310 (12)	−0.0119 (9)	−0.0087 (10)	0.0003 (9)
C18	0.0355 (12)	0.0278 (11)	0.0280 (12)	−0.0121 (10)	−0.0047 (10)	−0.0041 (9)
C19	0.0264 (11)	0.0287 (11)	0.0206 (10)	−0.0119 (9)	−0.0026 (8)	0.0002 (8)
C20	0.0210 (9)	0.0235 (10)	0.0215 (10)	−0.0083 (8)	−0.0084 (8)	0.0032 (8)
C21	0.0205 (9)	0.0220 (9)	0.0205 (10)	−0.0097 (8)	−0.0089 (8)	0.0039 (8)
C22	0.0193 (9)	0.0231 (10)	0.0194 (10)	−0.0091 (8)	−0.0076 (8)	0.0035 (8)
C23	0.0208 (9)	0.0272 (10)	0.0229 (10)	−0.0115 (8)	−0.0095 (8)	0.0059 (8)
C24	0.0257 (11)	0.0339 (12)	0.0255 (11)	−0.0146 (10)	−0.0070 (9)	0.0060 (9)
C25	0.0296 (11)	0.0371 (12)	0.0339 (13)	−0.0198 (10)	−0.0120 (10)	0.0128 (10)
C26	0.0317 (12)	0.0269 (11)	0.0421 (14)	−0.0159 (10)	−0.0151 (11)	0.0095 (10)
C27	0.0288 (11)	0.0241 (11)	0.0352 (13)	−0.0105 (9)	−0.0122 (10)	0.0029 (9)
C28	0.0213 (10)	0.0242 (10)	0.0268 (11)	−0.0097 (8)	−0.0113 (8)	0.0058 (8)
C29	0.0200 (9)	0.0235 (10)	0.0198 (10)	−0.0084 (8)	−0.0085 (8)	0.0026 (8)
C30	0.0210 (9)	0.0258 (10)	0.0185 (10)	−0.0104 (8)	−0.0079 (8)	0.0029 (8)
C31	0.0275 (11)	0.0306 (11)	0.0230 (11)	−0.0125 (9)	−0.0068 (9)	−0.0012 (9)
C32	0.0319 (12)	0.0409 (13)	0.0201 (11)	−0.0161 (11)	−0.0028 (9)	−0.0030 (9)
C33	0.0289 (11)	0.0440 (13)	0.0176 (10)	−0.0184 (11)	−0.0035 (9)	0.0031 (9)
C34	0.0189 (9)	0.0291 (10)	0.0180 (10)	−0.0101 (8)	−0.0085 (8)	0.0049 (8)
C35	0.0403 (14)	0.0471 (15)	0.0474 (16)	−0.0264 (13)	−0.0222 (13)	0.0224 (13)
C36	0.075 (2)	0.082 (3)	0.069 (2)	−0.035 (2)	−0.051 (2)	0.041 (2)
B1	0.0398 (16)	0.0427 (16)	0.0314 (15)	−0.0105 (14)	−0.0062 (13)	−0.0043 (12)
F1	0.203 (4)	0.0699 (18)	0.118 (3)	−0.039 (2)	−0.001 (3)	−0.0445 (17)
F2	0.0429 (13)	0.326 (5)	0.0545 (15)	−0.038 (2)	−0.0104 (11)	−0.029 (2)
F3	0.194 (4)	0.138 (3)	0.089 (2)	−0.106 (3)	−0.043 (2)	0.058 (2)
F4	0.0443 (11)	0.151 (2)	0.0487 (13)	0.0050 (13)	−0.0107 (10)	−0.0139 (14)

### Geometric parameters (Å, °)

**Table:**

Cu1—N2	2.0828 (19)	C13—H13A	0.9300
Cu1—N3	2.0628 (18)	C14—C15	1.382 (4)
Cu1—N6	2.013 (2)	C14—H14A	0.9300
Cu1—P2	2.1883 (6)	C15—C16	1.385 (3)
P2—C11	1.820 (2)	C15—H15A	0.9300
P2—C5	1.826 (2)	C16—H16A	0.9300
P2—C4	1.862 (2)	C17—C18	1.384 (3)
N1—C1	1.389 (3)	C17—H17A	0.9300
N1—C4	1.435 (3)	C18—C19	1.378 (3)
N1—H1A	0.8600	C18—H18A	0.9300
N2—C33	1.331 (3)	C19—C20	1.395 (3)
N2—C34	1.357 (3)	C19—H19A	0.9300
N3—C17	1.333 (3)	C20—C21	1.402 (3)
N3—C21	1.353 (3)	C20—C22	1.457 (3)
N4—C29	1.326 (3)	C21—C34	1.461 (3)
N4—C28	1.354 (3)	C22—C29	1.435 (3)
N5—C22	1.326 (3)	C23—C24	1.422 (3)
N5—C23	1.351 (3)	C23—C28	1.426 (3)
N6—C35	1.123 (3)	C24—C25	1.361 (3)
C1—C2	1.396 (3)	C24—H24A	0.9300
C1—C3^i^	1.400 (3)	C25—C26	1.413 (4)
C2—C3	1.390 (3)	C25—H25A	0.9300
C2—H2A	0.9300	C26—C27	1.363 (3)
C3—C1^i^	1.400 (3)	C26—H26A	0.9300
C3—H3A	0.9300	C27—C28	1.417 (3)
C4—H4A	0.9700	C27—H27A	0.9300
C4—H4B	0.9700	C29—C30	1.461 (3)
C5—C6	1.389 (3)	C30—C34	1.391 (3)
C5—C10	1.394 (3)	C30—C31	1.405 (3)
C6—C7	1.394 (4)	C31—C32	1.380 (3)
C6—H6A	0.9300	C31—H31A	0.9300
C7—C8	1.370 (5)	C32—C33	1.388 (3)
C7—H7A	0.9300	C32—H32A	0.9300
C8—C9	1.377 (4)	C33—H33A	0.9300
C8—H8A	0.9300	C35—C36	1.460 (4)
C9—C10	1.380 (4)	C36—H36A	0.9600
C9—H9A	0.9300	C36—H36B	0.9600
C10—H10A	0.9300	C36—H36C	0.9600
C11—C12	1.386 (3)	B1—F1	1.317 (4)
C11—C16	1.398 (3)	B1—F4	1.336 (4)
C12—C13	1.384 (4)	B1—F2	1.345 (4)
C12—H12A	0.9300	B1—F3	1.368 (4)
C13—C14	1.378 (4)		
			
N6—Cu1—N3	103.38 (8)	C16—C15—H15A	119.9
N6—Cu1—N2	106.93 (8)	C15—C16—C11	119.9 (2)
N3—Cu1—N2	80.51 (7)	C15—C16—H16A	120.1
N6—Cu1—P2	116.55 (6)	C11—C16—H16A	120.1
N3—Cu1—P2	130.67 (5)	N3—C17—C18	123.2 (2)
N2—Cu1—P2	111.75 (5)	N3—C17—H17A	118.4
C11—P2—C5	102.19 (9)	C18—C17—H17A	118.4
C11—P2—C4	99.92 (10)	C19—C18—C17	119.2 (2)
C5—P2—C4	104.86 (10)	C19—C18—H18A	120.4
C11—P2—Cu1	121.19 (7)	C17—C18—H18A	120.4
C5—P2—Cu1	108.62 (7)	C18—C19—C20	119.2 (2)
C4—P2—Cu1	117.95 (7)	C18—C19—H19A	120.4
C1—N1—C4	122.88 (19)	C20—C19—H19A	120.4
C1—N1—H1A	118.6	C19—C20—C21	117.91 (19)
C4—N1—H1A	118.6	C19—C20—C22	122.42 (19)
C33—N2—C34	117.71 (19)	C21—C20—C22	119.64 (18)
C33—N2—Cu1	129.25 (15)	N3—C21—C20	122.67 (19)
C34—N2—Cu1	112.74 (14)	N3—C21—C34	116.87 (18)
C17—N3—C21	117.82 (18)	C20—C21—C34	120.39 (18)
C17—N3—Cu1	128.72 (15)	N5—C22—C29	121.64 (18)
C21—N3—Cu1	113.35 (14)	N5—C22—C20	118.60 (18)
C29—N4—C28	117.24 (18)	C29—C22—C20	119.75 (18)
C22—N5—C23	117.20 (18)	N5—C23—C24	119.6 (2)
C35—N6—Cu1	171.4 (3)	N5—C23—C28	121.26 (19)
N1—C1—C2	119.23 (19)	C24—C23—C28	119.15 (19)
N1—C1—C3^i^	123.28 (19)	C25—C24—C23	119.9 (2)
C2—C1—C3^i^	117.44 (19)	C25—C24—H24A	120.0
C3—C2—C1	121.5 (2)	C23—C24—H24A	120.0
C3—C2—H2A	119.2	C24—C25—C26	120.8 (2)
C1—C2—H2A	119.2	C24—C25—H25A	119.6
C2—C3—C1^i^	121.04 (19)	C26—C25—H25A	119.6
C2—C3—H3A	119.5	C27—C26—C25	121.1 (2)
C1^i^—C3—H3A	119.5	C27—C26—H26A	119.5
N1—C4—P2	115.05 (15)	C25—C26—H26A	119.5
N1—C4—H4A	108.5	C26—C27—C28	119.7 (2)
P2—C4—H4A	108.5	C26—C27—H27A	120.1
N1—C4—H4B	108.5	C28—C27—H27A	120.1
P2—C4—H4B	108.5	N4—C28—C27	119.6 (2)
H4A—C4—H4B	107.5	N4—C28—C23	121.04 (19)
C6—C5—C10	118.6 (2)	C27—C28—C23	119.3 (2)
C6—C5—P2	124.84 (18)	N4—C29—C22	121.61 (19)
C10—C5—P2	116.59 (18)	N4—C29—C30	118.87 (18)
C5—C6—C7	119.9 (3)	C22—C29—C30	119.51 (18)
C5—C6—H6A	120.0	C34—C30—C31	118.03 (19)
C7—C6—H6A	120.0	C34—C30—C29	119.96 (18)
C8—C7—C6	120.8 (3)	C31—C30—C29	121.99 (19)
C8—C7—H7A	119.6	C32—C31—C30	119.0 (2)
C6—C7—H7A	119.6	C32—C31—H31A	120.5
C7—C8—C9	119.7 (2)	C30—C31—H31A	120.5
C7—C8—H8A	120.2	C31—C32—C33	118.9 (2)
C9—C8—H8A	120.2	C31—C32—H32A	120.6
C8—C9—C10	120.3 (3)	C33—C32—H32A	120.6
C8—C9—H9A	119.9	N2—C33—C32	123.5 (2)
C10—C9—H9A	119.9	N2—C33—H33A	118.3
C9—C10—C5	120.8 (3)	C32—C33—H33A	118.3
C9—C10—H10A	119.6	N2—C34—C30	122.87 (19)
C5—C10—H10A	119.6	N2—C34—C21	116.53 (18)
C12—C11—C16	119.2 (2)	C30—C34—C21	120.54 (18)
C12—C11—P2	119.89 (17)	N6—C35—C36	179.3 (3)
C16—C11—P2	120.85 (17)	C35—C36—H36A	109.5
C13—C12—C11	120.5 (2)	C35—C36—H36B	109.5
C13—C12—H12A	119.8	H36A—C36—H36B	109.5
C11—C12—H12A	119.8	C35—C36—H36C	109.5
C14—C13—C12	120.1 (3)	H36A—C36—H36C	109.5
C14—C13—H13A	119.9	H36B—C36—H36C	109.5
C12—C13—H13A	119.9	F1—B1—F4	111.9 (3)
C13—C14—C15	120.1 (2)	F1—B1—F2	110.7 (4)
C13—C14—H14A	120.0	F4—B1—F2	108.9 (3)
C15—C14—H14A	120.0	F1—B1—F3	103.6 (3)
C14—C15—C16	120.2 (2)	F4—B1—F3	110.1 (3)
C14—C15—H15A	119.9	F2—B1—F3	111.5 (3)

Symmetry codes: (i) −*x*, −*y*+1, −*z*.
